# Untargeted Plasma Metabolomics Unravels a Metabolic Signature for Tissue Sensitivity to Glucocorticoids in Healthy Subjects: Its Implications in Dietary Planning for a Healthy Lifestyle

**DOI:** 10.3390/nu13062120

**Published:** 2021-06-21

**Authors:** Nicolas C. Nicolaides, Maria-Konstantina Ioannidi, Eleni Koniari, Ifigeneia Papageorgiou, Anastasia Bartzeliotou, Amalia Sertedaki, Maria I. Klapa, Evangelia Charmandari

**Affiliations:** 1 Division of Endocrinology, Metabolism and Diabetes, First Department of Pediatrics, Νational and Kapodistrian University of Athens Medical School, ‘Aghia Sophia’ Children’s Hospital, 11527 Athens, Greece; nicolaidesnc@gmail.com (N.C.N.); helenia8@yahoo.it (E.K.); ifipap88@gmail.com (I.P.); aserted@med.uoa.gr (A.S.); 2 Division of Endocrinology and Metabolism, Center for Clinical, Experimental Surgery and Translational Research, Biomedical Research Foundation of the Academy of Athens, 11528 Athens, Greece; 3 Metabolic Engineering and Systems Biology Laboratory, Institute of Chemical Engineering Sciences, Foundation for Research and Technology-Hellas (FORTH/ICE-HT), 26504 Patras, Greece; m.k.ioannidi@gmail.com; 4 Department of Biology, University of Patras, 26500 Patras, Greece; 5 Department of Clinical Biochemistry, Νational and Kapodistrian University of Athens Medical School, ‘Aghia Sophia’ Children’s Hospital, 11527 Athens, Greece; anbartz@ymail.com

**Keywords:** tissue glucocorticoid sensitivity in healthy adults, dietary planning, glucocorticoid receptor, untargeted GC–MS metabolomics, blood plasma metabolic signature, precision medicine

## Abstract

In clinical practice, differences in glucocorticoid sensitivity among healthy subjects may influence the outcome and any adverse effects of glucocorticoid therapy. Thus, a fast and accurate methodology that could enable the classification of individuals based on their tissue glucocorticoid sensitivity would be of value. We investigated the usefulness of untargeted plasma metabolomics in identifying a panel of metabolites to distinguish glucocorticoid-resistant from glucocorticoid-sensitive healthy subjects who do not carry mutations in the human glucocorticoid receptor (*NR3C1*) gene. Applying a published methodology designed for the study of glucocorticoid sensitivity in healthy adults, 101 healthy subjects were ranked according to their tissue glucocorticoid sensitivity based on 8:00 a.m. serum cortisol concentrations following a very low-dose dexamethasone suppression test. Ten percent of the cohort, i.e., 11 participants, on each side of the ranking, with no *NR3C1* mutations or polymorphisms, were selected, respectively, as the most glucocorticoid-sensitive and most glucocorticoid-resistant of the cohort to be analyzed and compared with untargeted blood plasma metabolomics using gas chromatography–mass spectrometry (GC–MS). The acquired metabolic profiles were evaluated using multivariate statistical analysis methods. Nineteen metabolites were identified with significantly lower abundance in the most sensitive compared to the most resistant group of the cohort, including fatty acids, sugar alcohols, and serine/threonine metabolism intermediates. These results, combined with a higher glucose, sorbitol, and lactate abundance, suggest a higher Cori cycle, polyol pathway, and inter-tissue one-carbon metabolism rate and a lower fat mobilization rate at the fasting state in the most sensitive compared to the most resistant group. In fact, this was the first study correlating tissue glucocorticoid sensitivity with serine/threonine metabolism. Overall, the observed metabolic signature in this cohort implies a worse cardiometabolic profile in the most glucocorticoid-sensitive compared to the most glucocorticoid-resistant healthy subjects. These findings offer a metabolic signature that distinguishes most glucocorticoid-sensitive from most glucocorticoid-resistant healthy subjects to be further validated in larger cohorts. Moreover, they support the correlation of tissue glucocorticoid sensitivity with insulin resistance and metabolic syndrome-associated pathways, further emphasizing the need for nutritionists and doctors to consider the tissue glucocorticoid sensitivity in dietary and exercise planning, particularly when these subjects are to be treated with glucocorticoids.

## 1. Introduction

Glucocorticoids are steroid hormones synthesized in the adrenal cortex and released into the peripheral circulation, displaying ultradian and circadian rhythms, [1,2]. These endocrine molecules have several functions through binding to a ubiquitously expressed protein, the glucocorticoid receptor (GR), which acts as a ligand-induced transcription factor [3]. Within the target cell, the ligand-activated GR translocates into the nucleus and binds to the promoters of glucocorticoid target genes, increasing or decreasing their transcription rate [3,4]. Alternatively, the GR may regulate gene expression by interacting with other transcription factors, such as the activator protein-1 (AP-1), the nuclear factor-κB (NF-κB), and signal transducers and activators of transcription (STATs), influencing their transcriptional activity [3,4,5]. Further to their genomic actions, glucocorticoids may exert nongenomic actions, possibly through membrane-anchored GRs that activate kinase signaling pathways [6]. Through these numerous actions, glucocorticoids exert beneficial anti-inflammatory and immune-modulating effects, providing the basis for the therapeutic management of an ever-increasing number of disorders [7].

Tissue sensitivity to glucocorticoids has been influenced by an ever-increasing number of molecular, cellular, and tissue-associated factors. Specific polymorphisms or genetic defects in the *NR3C1* gene, which encodes the human glucocorticoid receptor (hGR), account for certain clinical phenotypes associated with glucocorticoid resistance or hypersensitivity [4,8,9]. Moreover, several receptor isoforms have been identified, further contributing to the complexity of tissue response to glucocorticoids [10]. Accumulating evidence suggests that the hGR “interactome” is enhanced by numerous novel partners, including RNA molecules (miRNA, non-coding RNA) [11,12].

In clinical practice, differences in glucocorticoid sensitivity among healthy subjects may influence the outcome and any adverse effects of glucocorticoid therapy. Due to their potent anti-inflammatory and immunosuppressive properties, glucocorticoids are used extensively to treat several allergic, inflammatory, hematologic, and lymphoproliferative disorders. However, a substantial number of patients with the above disorders may not respond adequately to glucocorticoids because of tissue-specific glucocorticoid resistance, while other subjects may rapidly develop symptoms and signs of hypercortisolism. The above differences in the response to the same dose and duration of glucocorticoid therapy among subjects have been attributed to individual variations in glucocorticoid sensitivity [8]. Although the various actions of glucocorticoids in many biological processes and the side effects of synthetic glucocorticoids are well-recognized, it still remains difficult to detect individual variations in tissue responsiveness to glucocorticoids among healthy adults in order to adjust the therapeutic dose of synthetic glucocorticoids appropriately when they need treatment with glucocorticoids. In addition, glucocorticoids have been associated with weight gain, which can be substantially increased when combined with a high-fat diet [13], while dietary advanced glycation end-products (AGEs) were recently shown to induce glucocorticoid resistance [14]. Therefore, the identification of novel biomarkers and the development of fast and accurate methods for distinguishing healthy subjects as most glucocorticoid-sensitive or most glucocorticoid-resistant remains very important, not only for appropriately selecting a course of glucocorticoid treatment in inflammatory diseases but also in designing a dietary and exercise plan. Accurate assessment of the tissue glucocorticoid sensitivity of an individual can play an important role in these decisions.

In the era of personalized and precision medicine (PPM), high-throughput biomolecular (omic) analyses of blood and biofluids are pivotal because they provide specific information on the pathogenesis, diagnosis, prognosis, or progression of a disease, and facilitate the design and development of novel therapeutic strategies. Peripheral blood is one of the most easily accessible human tissues in a noninvasive way, and its analysis can elucidate the (patho)physiologic state of any organism. Untargeted plasma metabolomics quantifies and analyzes in a multivariate way the concentration profile of the plasma metabolites. Hence, it provides a comprehensive functional readout of a particular (patho)physiologic state, while reflecting events occurring downstream of gene expression, representing a direct link to phenotype [15,16]. In view of molecular physiology as an interconnecting network of biomolecular networks, even subtle differences in a molecular quantity may result in substantial outcomes within the framework of changes in other interacting molecules according to their relative position and biological role [17]. In this context, metabolomics is emerging as a valuable tool to study human (patho)physiology, opening a new field to elucidate health and disease progression and identify disease-specific metabolic signatures to diagnose and assess disease risks and appropriately design therapeutic treatments. In the same context, metabolomics can play an important role in dietary and exercise planning within therapeutic strategies and also to establish a healthy lifestyle, in general [18].

Metabolomics can and should be part of clinical practice as a component of the clinical chemistry laboratory [19]. Its advantages over the other omics include its lower cost, no need for special technological platforms, and the use of classical analytical chemistry equipment, most being part of a clinical chemistry laboratory: nuclear magnetic resonance (NMR) spectroscopy and/or mass spectrometry (MS) integrated with gas or liquid chromatography (GC or LC) [17,19]. It is worth mentioning that untargeted metabolomics has not yet been used to investigate the metabolic alterations and implications associated with the individual variations in tissue glucocorticoid sensitivity in human subjects. In this context, the aim of our study was to search for a metabolic signature that could be used to distinguish most glucocorticoid-resistant from most glucocorticoid-sensitive healthy adult subjects, who do not carry mutations in the human glucocorticoid receptor (*NR3C1*) gene, using untargeted plasma GC–MS-based metabolomics, which monitors mainly the primary metabolism. To this end, we applied a very low-dose dexamethasone suppression test that had been explicitly designed and used to assess the tissue glucocorticoid sensitivity in healthy subjects [8,20] to rank a gender-balanced cohort of 101 young (mean age 26.5 ± 5 years) healthy subjects. The eleven (11) individuals, i.e., 10% of the cohort, on each side of the ranking, who did not carry mutations in the human glucocorticoid receptor (*NR3C1*) gene, were selected as the most glucocorticoid-sensitive and the most glucocorticoid-resistant of this cohort. These two “extremes” of the 101-participant group were deemed as most appropriate to be analyzed and compared with respect to their blood plasma metabolic profile to reveal any associated metabolic differences in this first study of this type, which could be further explored in larger clinical contexts.

## 2. Materials and Methods

### 2.1. Healthy Subject Cohort Description—Selection of the Most Glucocorticoid-Sensitive and Most Glucocorticoid-Resistant Subgroups

One hundred one healthy subjects (*n* = 101; 50 males and 51 females) of a mean age (±SD) 26.5 (±5) years, who were not taking any medications, including oral contraceptives for the women participants, were recruited prospectively. The study was set up and applied according to the published protocol of a very low-dose dexamethasone suppression test, which had been explicitly developed to study interindividual variation in tissue glucocorticoid sensitivity in healthy subjects [8,20]. More specifically, all participants were given a very low-dose (0.25 mg) of oral dexamethasone at midnight, and serum cortisol (261.8 ± 206.9 nmol/L) and plasma ACTH (16.1 ± 12.2 pg/mL) concentrations were determined at 8:00 a.m. the following morning. The very low dexamethasone dose was selected, and the protocol was designed to detect very mild differences owing to interindividual variation in tissue glucocorticoid sensitivity among healthy participants. As in these studies the cortisol concentrations of all participants are within the normal healthy range, the application of a cortisol cut-off was out of context. Rather, the participants were rank-ordered according to their 8:00 a.m. serum cortisol concentrations and the two “extreme” subgroups were, respectively, selected as the most glucocorticoid-sensitive and the most glucocorticoid-resistant of the particular cohort.

In our study, we selected 10% of the 101 healthy subject cohort, i.e., 11 participants, on each side of the ranking, who did not carry mutations in the *NR3C1* gene, to participate in the blood plasma metabolomic study. More specifically, the 11 individuals with the lowest cortisol concentrations and the 11 with the highest cortisol concentration were selected as the most glucocorticoid-sensitive (S) and the most glucocorticoid-resistant (R), respectively, of the original cohort [(mean serum cortisol concentrations ± SD: 34.4 ± 15 nmol/L in the S participants vs. 622.4 ± 93.7 nmol/L in the R participants, *p* < 0.001); (mean plasma ACTH concentrations ± SD: 2.8 ± 2.4 pg/mL in the S participants vs. 31.6 ± 10.6 pg/mL in the R participants, *p* < 0.001)] (Table 1). These two subgroups of 11 individuals each participated in further analyses, and one month after the very low-dose dexamethasone suppression test, DNA, RNA, and plasma samples were collected for the genetic, biochemical, and metabolomic analyses of the study.

### 2.2. Ethical Considerations

The study was approved by the “Aghia Sophia” Children’s Hospital Committee on the Ethics of Human Research (Approval Number: EB-PASCH-MoM: 13/02/2013, Re: 1490-21/01/2013). All participants provided written informed consent before participating in the study.

### 2.3. Sample Collection

Blood samples (5 mL) were obtained from the study participants at 8:00 a.m. in EDTA-containing tubes and centrifuged immediately after collection. Blood plasma samples were stored at −80 °C until further analysis. Aliquots of 150 μL were collected for each blood plasma sample and two aliquots per sample were shipped in dry ice to the metabolomic analysis laboratory.

### 2.4. Assays

The standard hematologic parameters were measured using the ADVIA 2110i analyzer (Roche Diagnostics, GmbH, Mannheim, Germany). Glucose, triglycerides (TG), total cholesterol (t-CHOL), and high-density lipoprotein cholesterol (HDL-C) were quantified using the ADVIA 1800 Siemens analyzer (Siemens Healthcare Diagnostics, Tarrytown, NY, USA). Lipoprotein (a) (Lp(a)) as well as apolipoproteins A1 (ApoA1) and B (ApoB) were quantified by latex particle-enhanced immunonephelometric assays on the BN ProSpec nephelometer (Dade Behring, Siemens Healthcare Diagnostics, Liederbach, Germany).

Ferritin, insulin, LH, FSH, and estradiol were quantified using automated electrochemiluminescence immunoassays “ECLIA” (Analyzer Cobas e411, Roche Diagnostics, GmbH, Mannheim, Germany). TSH, free (f) T4, anti-TPO, anti-TG, androstenedione, DHEA-S, ACTH, cortisol, IGF-I, IGFBP-3, and high-sensitivity C-reactive protein (hs-CRP) concentrations were determined using automated chemiluminescence immunoassays on an IMMULITE 2000 Immunoassay System (Siemens Healthcare Diagnostics Products Ltd., Frimley, Camberley, Surrey, UK). Total 25-hydroxyvitamin D (25-OH Vitamin D) concentrations were determined using automated electrochemiluminescence immunoassays (Modular Analytics E170 analyzer, Roche Diagnostics, GmbH, Mannheim, Germany). HbA1C was measured using reversed-phase cation exchange high-performance liquid chromatography (HPLC) on an automated glycohemoglobin analyzer HA-8160 (Arkray, Kyoto, Japan). The applied quantification procedures are the standard used in clinical practice for defining the glucocorticoid sensitivity of treated individuals.

### 2.5. Sequencing of the NR3C1 Gene

The Maxwell 16 instrument for automated DNA extraction was used to isolate genomic DNA from peripheral leukocytes (Promega Corp., Madison, WI, USA). Sequencing of the *NR3C1* gene (NM_000176.3) was performed as previously described [21]. Specifically, the *NR3C1* gene coding regions and their flanking sequences on chromosome 5 (GRCh37:NR3C1-201 ENST00000343796.2) analyzed in this work were presented and numbered relative to coordinates of the *NR3C1* gene: Exon 2: 142780516–142779199, Exon 3: 142693835-142693511, Exon 4: 142689849–142689542, Exon 5: 142680396–142679927, Exon 6: 142678424–142678207, Exon 7: 142675319–142674851, Exon 8: 142662436-142662012, Exon 9 and 3′UTR: 142661729–142661302.

### 2.6. Metabolic Profile Acquisition, Normalization & Filtering

Polar and semipolar metabolite extraction and untargeted GC–MS metabolic profile acquisition protocols were followed as previously described [17,19,22,23]. More specifically in this study, 30 μg ribitol (Alfa Aesar, Heysham, UK) and 30 μg (U-^13^C)-D-glucose (Cambridge Isotope Laboratories, Cambridge, MA, USA) were added as internal standards in each 150 μL plasma aliquot. The dried extracts were derivatized to their (MeOx)TMS derivatives through reaction first with 50 μL of 20 mg/mL methoxyamine hydrochloride (MeOx-HCL) (Alfa Aesar, Thermo Fisher (Kandel) GmbH, Kandel, Germany) in pyridine (Carlo Erba Reagents, Cornaredo (MI), Italy) for 90 min, followed by a reaction with 100 μL of N-methyl-trimethylsylil-trifluoroacetamidine (MSTFA) (Alfa Aesar, Thermo Fisher (Kandel) GmbH, Kandel Germany) at 40 °C for at least 6 h. The GC–MS analysis was performed using the Saturn 2200 GC-(ion trap)MS (formerly Varian Inc., currently Bruker/Agilent, Santa Clara, CA, USA). The sequence of sample analysis was randomized, and the profile of each aliquot was measured at least thrice at different derivatization times. Peak identification and quantification were based on the commercial NIST and our in-house MESBL peak library containing more than 700 reviewed peaks annotated and classified following the standards previously described [24]. Appropriate metabolomic data validation, normalization, and filtering were carried out based on the criteria described and justified in [19,22,23] using the relevant module of M-IOLITE, the GC–MS metabolomic analysis streamlining software suite of our group (http://miolite2.iceht.forth.gr) [24]. The metabolite derivative relative peak areas (RPAs) were estimated with respect to the 319 marker ion of the internal standard ribitol. The glucose RPA was estimated from the sum of glucose-MeOx1, glucopyranose 1, and glucopyranose 2 derivative RPAs. After normalization and filtering, each metabolic profile comprised 50 metabolite RPAs. The normalized metabolic profile of each aliquot was estimated from the mean of the normalized profiles of its technical replicates and the mean aliquot profile of each sample was used in further analyses. The final normalized metabolomic dataset considered in multivariate statistical analysis is provided in Supplementary Table S1, containing all information about the peak annotation confidence level, too.

### 2.7. Multivariate Analysis of the Metabolomic Dataset

Principal component analysis (PCA) and significance analysis for microarrays (SAM) algorithms were used as implemented in version 4.9.0 of the omic data analysis software TM4/MeV [25,26]. In the multivariate significance analysis method SAM, the metabolites, the abundance of which were identified as significantly higher or lower in a set of metabolic profiles compared to one another, will be, respectively, referred to as “positively” or “negatively” significant metabolites of the particular comparison for a selected significance threshold associated with a false discovery rate (FDR-median). The computational analysis of untargeted GC–MS metabolomic data followed in this study has been previously described [23].

### 2.8. Statistical Analyses

Results are presented as mean ± standard deviation (SD). Normality was tested graphically according to histograms and Q–Q plots to determine whether or not to use parametric methods for the sample data analysis. The associations between variables and participant groups were evaluated by Student’s t-tests or the Mann–Whitney U tests for independent samples. All statistical tests were two-sided and performed at a 0.05 significance level. Data analyses were performed using the SPSS (Chicago, IL, USA) statistical package version 24.0.

## 3. Results

### 3.1. Clinical Characteristics, Biochemical and Endocrinologic Parameters of the Participants

The clinical characteristics, as well as the biochemical and endocrinologic parameters of the 22 healthy participants at the time of the very low-dose dexamethasone suppression test and one month later are shown in Table 1 and Table 2, respectively. No statistically significant difference in the endocrinologic parameters was identified between the S and R groups (*p* value > 0.05). We noted that considering the reproductive cycle of the women participants, gonadotropin and sex steroid concentrations in women were determined between the 3rd and 5th day of the menstrual cycle.

### 3.2. NR3C1 Gene Sequencing Revealed No Polymorphisms or Mutations in the 22 Subjects

To investigate whether any genetic defects or polymorphisms in the *NR3C1* gene could explain this variation in tissue glucocorticoid sensitivity, the protein-expressing region and the intron/exon junctions were PCR-amplified and sequenced bidirectionally. No genetic defects or polymorphisms were detected in the *NR3C1* gene of the 22 subjects.

### 3.3. Metabolic Profiling Analysis

Multivariate statistical analysis of the 22 metabolic profiles indicated individual R11_m as having a substantially different plasma metabolic profile from all others. Interestingly, also, while the participant (male) had been characterized as glucocorticoid-resistant based on the cortisol measurement of the dexamethasone suppression test, his sample clustered with the sensitive profiles (Supplementary Figure S1). To avoid skewing the results, this particular profile was excluded from further analysis. Reviewing this participant’s medical record, it became evident that he had undertaken excessive exercise a few days before the blood plasma sampling (a month after the very low-dose dexamethasone suppression test). Interestingly, the discriminatory profile of R11_m became evident in its metabolomic data as no significant difference was observed in their biochemical and endocrinological parameters (see Supplementary Table S2 replicating Table 2 for S and R groups but in the absence of R11_m), supporting the sensitivity of the metabolic profile to identify such differences.

Subsequently, we searched for specific discriminatory metabolites between the S and R groups using multivariate significance analysis based on the SAM method. The analysis indicated that the total abundance of the 50 analyzed metabolites was on average lower in the S compared to the R group, implying that the relevant pathways and metabolic processes are of lower activity/flux in the S compared to the R group. In this context of the total abundance decrease, for the strictest significance threshold providing results, the SAM method identified 19 metabolites with significantly lower abundances (“negatively significant”) in the S compared to the R subjects, but no metabolite crossed the positive significance threshold (Table 3, Supplementary Figure S2). The negatively significant metabolites include lipids, i.e., the polyunsaturated (PUFA) and saturated fatty acid (SFA), respectively, linoleic and octadecanoic (stearic) acid, the monoglycerides (MAGs) glycerol monostearate and glycerol monopalmitate (1-monopalmitin), erythritol, myo-inositol, 2-hydroxybutyrate, and the amino acids glycine, serine, threonine, and glutamate. On the other side, an unknown sugar pyranose putatively annotated as galactopyranose, sorbitol, lactate, and glucose were in this order of decreasing significance the four metabolites identified with a higher abundance in the S compared to the R subjects, just below the positive significance threshold (Supplementary Figure S2). It is important to consider these metabolites as they enhance the perspective of the metabolic physiology that can be obtained by the combined analysis of interconnected metabolites in the context of metabolic pathways. In addition, the mean relative composition of the 50 metabolites in these four metabolites is larger in the S compared to R group, but they do not “cross” the significance threshold with respect to the actual abundance in the context of the overall abundance decrease of the 50 metabolites in the S vs the R group. Finally, the galactopyranose and sorbitol exhibited a characteristically larger abundance in the first four most sensitive individuals of the cohort, differentiating them from the rest and in decreasing order from the first to the fourth S individual (Supplementary Table S1).

## 4. Discussion

We performed untargeted plasma metabolomics in healthy subjects with marked differences in tissue glucocorticoid sensitivity based on their response to a very low-dose dexamethasone suppression test. Multivariate significance analysis of the profiles indicated a set of metabolites, the difference in the abundance of which could be discriminatory of individuals belonging to the two groups. Sorbitol appeared among the metabolites, with higher abundances in the glucocorticoid-sensitive individual profiles. Sorbitol accumulation in the blood indicates a higher rate of the polyol pathway in the S compared to the R subjects, a metabolic route connected to hyperglycemic conditions and oxidative stress [27]. Τhis result concurs with the observed higher concentration of glucose and lactate in the S compared to the R group, which implies a higher Cori cycle rate in the S compared to the R subjects after overnight fasting. This phenomenon has been associated with metabolic syndrome and insulin resistance conditions [28].

Moreover, we found that the S subjects had statistically significantly lower abundances of the linoleic (PUFA) and octadecanoic/stearic (SFA) acids, as well as the monoglycerides glycerol-monostearate and glycerol-monopalmitate. Decreased linoleic acid concentration has been associated with hypertension and cardiovascular disease [29]. These findings in combination imply a lower rate of fat mobilization after overnight fasting in the S compared to the R subjects. This phenomenon has been associated with decreased expression of the hormone-stimulated enzymes regulating the fat mobilization process [30] and has been related to insulin resistance and obesity. Fatty acids related to triglyceride metabolism (e.g., palmitic, stearic, linoleic) have been associated with glucocorticoid action in two recent untargeted metabolomic studies of the dose-dependent effect of glucocorticoid treatment of glucocorticoid-dependent disorders [31,32].

Finally, the S group exhibited lower abundances of plasma glycine, serine, and threonine compared to the R group. This is the first study linking increased glucocorticoid sensitivity to glycine, serine, and threonine metabolism based on the collective profile of these amino acids. Threonine was identified as differential in an untargeted metabolomic study of the dose-dependent effect of glucocorticoid treatment of congenital adrenal hyperplasia [31]. Lower plasma levels of these amino acids may imply their higher uptake rate from the tissues toward higher inter-tissue rates of cytosolic one-carbon metabolism associated with mitochondrial deficiency and oxidative stress [33,34]. We hypothesize that the activation of one-carbon metabolism, observed in the glucocorticoid-sensitive subjects, could be a counter-regulatory mechanism to reduce oxidative stress, to increase energy production, as well as to provide methyl groups for epigenetic modifications [35,36]. Moreover, by upregulating glucose consumption, this metabolic pathway could be further used to reduce the stress-increased glucose concentrations and, therefore, to avoid any chronic hyperglycemia complications [37].

The acquired results agree with current knowledge about the differences between glucocorticoid-sensitive and glucocorticoid-resistant healthy subjects owing to polymorphisms of the *NR3C1* gene given that the former are known to exhibit an unfavorable cardiometabolic profile compared to their normal counterparts [38,39]. Indeed, carriers of the N363S polymorphism of the *NR3C1* gene, who have higher sensitivity to glucocorticoids, have a higher waist-to-hip ratio, increased body mass index (BMI), elevated concentrations of cholesterol and triglycerides, lower bone mineral density, and higher prevalence of coronary artery disease independent of weight gain [40,41,42]. In addition to the N363S polymorphism, the BclI polymorphism of the *NR3C1* gene has also been associated with increased glucocorticoid sensitivity [43]. Subjects with this polymorphism are obese and more susceptible to chronic diseases, such as hypertension, bronchial asthma, and mood disorders [44,45]. Furthermore, one point mutation in the *NR3C1* gene has been associated with primary generalized glucocorticoid hypersensitivity. This was a heterozygous guanine to cytosine (G→C) substitution at nucleotide position 1201 in exon 2 of the *NR3C1* gene, which led to an aspartic acid (D) to histidine (H) substitution at amino acid position 401 in the N-terminal domain of the receptor [46]. This mutation was identified in a 43-year-old female who displayed all cardinal manifestations of metabolic syndrome (visceral obesity, hypertriglyceridemia, hypercholesterolemia, type 2 diabetes, and hypertension) [46]. Moreover, there have been cases of transient generalized glucocorticoid hypersensitivity in which no genetic defects in the *NR3C1* gene were discovered, suggesting that other unknown factors (i.e., viral proteins) might trigger the activation of the hypothalamic–pituitary–adrenal (HPA) axis in these patients [47,48,49]. Finally, patients with Cushing’s syndrome display a worse metabolic profile due to hypercortisolism [50,51]. However, the participants in our study were all healthy and did not have any genetic defects or polymorphisms in the *NR3C1* gene, indicating that other factors in the glucocorticoid signaling pathway might influence tissue sensitivity to glucocorticoids and contribute to this distinct metabolic phenotype.

Our study succeeded in identifying a metabolic signature that might be used to differentiate glucocorticoid-resistant from glucocorticoid-sensitive healthy subjects based on their metabolic profiling. This is a first pilot metabolomic study for the investigation of tissue glucocorticoid sensitivity in healthy subjects and our results can form the basis for future larger studies. Indeed, as the sample size of our study was relatively small, comprising 10% of the initially studied and screened (*n* = 101) participants in each of the S and R groups (22 subjects in total), further and larger studies are undoubtedly needed to validate and provide convincing explanations about the differences observed between the glucocorticoid-sensitive and glucocorticoid-resistant subjects. In the limitations of our study, we may also consider the use of immulite instead of HPLC for determining serum cortisol concentrations as we carried out the standard procedure that we normally follow in clinical practice. The purpose of our study was to directly compare the information obtained by the standard in clinical practice procedures for determining glucocorticoid sensitivity, which is currently used to decide the appropriate glucocorticoid treatment, with that acquired from metabolic profiling analysis. Finally, we did not determine the dexamethasone concentrations of the participants following the very-low dexamethasone suppression test because we designed our study according to similar studies that determined glucocorticoid sensitivity in healthy subjects, such as the study by Donn et al., who identified a new glucocorticoid sensitivity-determining gene using gene expression profiling [20]. Furthermore, in most published studies that use dexamethasone suppression tests, researchers have not routinely determined dexamethasone concentrations in their subjects [52,53,54,55].

From the clinical point of view, the results of the present study—if validated by further larger studies—hold a particular significance as a panel of differential between the S and R groups could be used for the development of diagnostic regression models that could assist in classifying an individual based on his/her tissue glucocorticoid sensitivity and appropriately adjusting the dose of synthetic glucocorticoids in patients with glucocorticoid-dependent disorders in order to achieve better clinical outcome and fewer adverse effects [7]. However, it should be noted that the metabolic physiology might be altered in the context of these disorders; therefore, our metabolomic signature might not be directly informative and specific studies should be carried out to identify the effect of interindividual variation of tissue glucocorticoid sensitivity on the efficacy of the therapeutic treatment. Moreover, our results support the connection of glucocorticoid sensitivity with metabolic processes associated with insulin resistance, metabolic syndrome, inflammation, and obesity, making the assessment and classification of an individual with respect to his/her glucocorticoid sensitivity a pivotal part of dietary and exercise planning both under pathophysiological conditions as part of an optimized therapeutic treatment and also for the adoption of a healthy lifestyle. Based on the acquired results, the most glucocorticoid-sensitive subjects may be more careful in their eating and exercise habits, emphasizing on a long-term healthy lifestyle of a low-fat diet and regular physical exercise.

The consumption of high-fat and high-sugar foods (“comfort foods”) is proportional to circulating glucocorticoids and/or glucocorticoid sensitivity as indicated by the fact that patients with Cushing’s disease choose foods containing high fat compared to subjects with normal glucocorticoid concentrations [56]. Studies in rodents that investigated whether prior metabolic stress (restraint or cold stress) influenced the preference for “comfort foods” and modulated the subsequent HPA axis response showed that rodents preferred “comfort” foods to standard chow. Their provision on these “comfort” foods led to a reduction of the degree of the stressor-activated sympathetic responses and reduced the basal concentrations of corticotropin-releasing factor/hormone (CRF) in the hypothalamus [57]. Glucocorticoids enhance feeding behavior by altering the levels of neuropeptide Y (NPY), a key orexigenic neurotransmitter associated with food consumption and deposition of adipose tissue [58]. Clinically, this is very relevant, as it suggests that healthy subjects who are most glucocorticoid-sensitive display higher risk for developing metabolic complications if they systematically prefer a high-fat diet, particularly if they need to take exogenous glucocorticoid therapy. On the other hand, regular physical exercise is used widely to address many cardiometabolic conditions due to its beneficial actions with several organs, including muscle, adipose tissue, liver, and bone. While increased glucocorticoid sensitivity induces insulin resistance, physical exercise increases insulin sensitivity and reduces the expression of both GR and 11-β-HSD1 within the insulin-sensitive organs (skeletal muscle, adipose tissue, and liver), ultimately reducing tissue exposure to glucocorticoids [13,59]. On the other hand, in the context of the one identified case in our cohort, it appears that excessive exercise can induce substantial stress to the body, modifying the glucocorticoid sensitivity status of the individual. This is an observation in clear need for further specific investigation.

We speculate that pre-existing epigenetic alterations might influence the expression of genes, thereby regulating several important metabolic pathways [21]. Furthermore, we cannot predict whether these metabolic alterations might be prevented in the future through a healthy lifestyle. Sex-specific differentiations should also be investigated. Supporting the metabolic findings with proteomic and lipidomic data would be of importance to further enhance physiological signatures leading to a more accurate determination of the glucocorticoid sensitivity of each individual.

## Figures and Tables

**Table 1:** Clinical characteristics, serum cortisol and plasma ACTH concentrations of the most glucocorticoid-sensitive (S) and most glucocorticoid-resistant (R) healthy subjects at the time of the very-low dexamethasone suppression test.

	Sample Code	Sex	Weight (kg)	Height (m)	BMI (kg/m^2^)	Cortisol (nmol/L)	ACTH (pg/mL)
**Glucocorticoid-Sensitive (S)**	1	F	58	1.64	21.6	18.6	<1.0
	2	F	62	1.75	20.2	22.2	1.4
	3	M	70	1.77	22.3	23.1	6.2
	4	F	45	1.50	20,0	24.5	<1.0
	5	M	70	1.85	20.5	26.2	2.9
	6	F	55	1.64	20.4	32.3	<1.0
	7	F	48	1.57	19.5	34.2	5.1
	8	M	80	1.78	25.2	36.1	<1.0
	9	M	70	1.82	21.1	39.7	2.0
	10	M	52	1.71	17.8	51.3	<1.0
	11	M	81	1.87	23.2	69.5	7.6
	**Mean Value ± SD**				**21.1 ± 2.0**	**34.4 ± 15**	**2.8 ± 2.4**
**Glucocorticoid-Resistant (R)**	1	F	52	1.59	20.6	834.0	35.3
	2	F	56	1.68	19.8	720.9	38.1
	3	F	59	1.55	24.6	690.8	46.0
	4	M	93	1.86	26.9	644.2	42.2
	5	M	53	1.68	18.8	599.0	32.8
	6	F	47	1.54	19.8	597.9	23.7
	7	F	59	1.70	20.4	579.4	39.9
	8	F	58	1.65	21.3	565.3	16.1
	9	F	58	1.7	20.1	556.2	29.9
	10	M	70	1.72	23.7	537.4	30.9
	11	M	77	1.88	21.8	520.6	12.4
	**Mean Value ± SD ^(1)^**				**21.6 ± 2.5**	**622.4 ± 93.7**	**31.6 ± 10.6**

BMI: body mass index; ACTH: adrenocorticotropic hormone. **^(1)^** The *p*-value for BMI, cortisol and ACTH were, respectively, *p* = 0.797, *p* < 0.001, *p* < 0.001.

**Table 2:** Clinical characteristics, biochemical and endocrinologic parameters of the most glucocorticoid-sensitive (S) and most glucocorticoid-resistant (R) healthy subjects one month after the very-low dexamethasone suppression test.

	Glucocorticoid-Sensitive (S)	Glucocorticoid-Resistant (R)	*p* Value
Age (years)	25.3 ± 3.9	27.5 ± 6.7	0.478
Weight (kg)	62.8 ± 12.3	62 ± 13.2	0.847
Height (cm)	1.7 ± 0.1	1.7 ± 0.1	0.519
BMI (kg/m^2^)	21.1 ± 2.0	21.6 ± 2.5	0.797
25-Hydroxy-Vitamin D (ng/mL)	16.0 ± 7.9	14.0 ± 8.5	0.652
ACTH (pg/mL)	33.2 ± 18.8	27.6 ± 15.4	0.519
Androstenedione (ng/mL)	2.9 ± 0.9	3.2 ± 1.2	0.502
Anti-TG (IU/mL)	20 ± 0.0	20 ± 0.0	0.999
Anti-TPO (IU/mL)	10.4 ± 0.7	11.1 ± 2.6	0.652
ApoA1 (mg/dL)	158.4 ± 8.0	167.6 ± 15.0	0.237
ApoB (mg/dL)	75.5 ± 14.4	71.4 ± 7.7	0.515
Total Cholesterol (mg/dL)	157.4 ± 16.9	156. ± 15.0	0.965
Cortisol (nmol/L)	638.2 ± 155.3	523.7 ± 280.0	0.270
DHEAS (μg/dL)	238.6 ± 146.0	248.6 ± 115.0	0.562
FSH (mUI/mL)	5.2 ± 2.7	4.0 ± 2.3	0.300
FT4 (ng/dL)	1.1 ± 0.1	1.1 ± 0.1	0.261
Glucose (mg/dL)	73.2 ± 6.3	74.7 ± 13.6	0.965
HDL (mg/dL)	49.5 ± 7.0	52.9 ± 8.1	0.315
IGFBP-3 (μg/mL)	5.3 ± 1.0	5.2 ± 1.2	0.562
IGF-I (ng/mL)	259.2 ± 79.5	251.4 ± 66.8	0.699
Insulin (μUI/mL)	6.7 ± 2.7	13.7 ± 1	0.116
LDL (mg/dL)	90.7 ± 17.8	87.6 ± 13.5	0.762
LH (mUI/mL)	10.1 ± 14.9	6.4 ± 2.3	0.699
Lp(a) (mg/dL)	21.8 ± 37.4	25.8 ± 27.3	0.460
Prolactin (ng/mL)	24.9 ± 8.8	21.5 ± 9.1	0.193
PTH (pg/mL)	34.1 ± 15.2	38.5 ± 17.9	0.562
SHBG (nmol/L)	65.1 ± 27.9	46.2 ± 15.3	0.175
T3 (ng/dL)	102.3 ± 27.6	102.0 ± 23.8	0.982
Triglycerides (mg/dL)	69.4 ± 30.0	74.2 ± 16	0.315
TSH (μUI/mL)	2.8 ± 0.9	2.0 ± 1.1	0.101

The parameters for the two groups are expressed as mean ± SD (*n* = 11). ACTH: adrenocorticotropic hormone, Anti-Tg: thyroglobulin antibodies, Anti-TPO: thyroid peroxidase antibodies, BMI: body mass index; DHEAS: dehydroepiandrosterone sulfate, FSH: follicle-stimulating hormone, FT4: free thyroxine, IGF-I: insulin-like growth factor-I, IGFBP-3: insulin-like growth factor-binding protein 3, LH: luteinizing hormone, PTH: parathormone, SHBG: sex hormone-binding globulin, T3: triiodothyronine, TSH: thyroid-stimulating hormone.

**Table 3:** The blood plasma metabolites identified with significantly lower abundances in the most glucocorticoid-sensitive (S) compared to the most glucocorticoid-resistant (R) healthy subjects based on the multivariate significance analysis SAM method, as implemented in the TM4/MeV software. The metabolites are shown in decreasing significance based on the SAM curve shown in Figure S2.

**Negatively Significant Metabolites in the S vs. the R Group in Decreasing Significance for FDR-Median = 23.5% (or 4.5 Metabolites)** ^(1)^
Octadecanoic (stearic) acid
2. Un_0063 ^(2)^
3. Glycerol 1-palmitate
4. 9,12-octadecadienoic acid (Z,Z) (linoleic acid)
5. Un_ 0180 (sugar, putatively)
6. Glycine
7. Un_0130 (sugar pyranose putatively)
8. Myo-inositol
9. Un_0253 (sugar acid putatively)
10. Threonine
11. Serine
12. Un_0134 (sugar pyranose putatively)
13. Erythritol
14. Un_0185
15. Un_0254 (steroid putatively)
16. 2,3-Dihydroxypropyl octadecanoate (glycerol monostearate)
17. Urea
18. Glutamate ^(3)^
19. 2-hydroxybutanoic acid

^(1)^ The particular FDR-median is the lowest for which significant metabolites were identified; it corresponds to a significance threshold δ (delta) equal to 0.508 (see Figure S2). ^(2)^ The identifiers for the unknown metabolites are as archived in the peak library of our group; the full normalized metabolomic dataset is provided in Supplementary Table S1. ^(3)^ The glutamate abundance is based on its pyroglutamate derivative measurement (Kanani et al., 2007).

## Data Availability

The original data generated, analyzed, and discussed in the study are included in the article/supplementary material; further inquiries can be directed to the corresponding authors.

## Supplementary Materials

The following are available online at https://www.mdpi.com/article/10.3390/nu13062120/s1, Figure S1: The PCA graph of the metabolomic dataset of all 22 samples, Figure S2: The SAM curve of the metabolic profile data of the S (Group B) compared to the R (Group A) groups, Table S1: The normalized blood plasma metabolomic dataset of the 22 participants used in multivariate statistical analysis, Table S2: Clinical characteristics, biochemical and endocrinological parameters of the most glucocorticoid sensitive (S) and most glucocorticoid resistant (R), excluding R11_m, healthy subjects one month after the very-low dexamethasone suppression test.
