# Supplementary material for: Untargeted Plasma Metabolomics Unravels a Metabolic Signature for Tissue Sensitivity to Glucocorticoids in Healthy Subjects: Its Implications in Dietary Planning for a Healthy Lifestyle
Source: Nutrients. 2021 Jun 21;13(6):2120. doi: 10.3390/nu13062120 (PMC8234096; doi:10.3390/nu13062120)
Supplement: Supplementary file 1 [file nutrients-13-02120-s001.zip › nutrients-1214555-supplementary.pdf]

nutrients

Nicolaides et al_Supplementary Material

Table of Contents

Supplementary Table 1: The normalized blood plasma metabolomic dataset of the 22 participants used in multivariate statistical analysis……………………………..………………………………….2

Supplementary Figure 1: The PCA graph of the metabolomic dataset of all 22 samples……..........3

Supplementary Table 2: Clinical characteristics, biochemical and endocrinological parameters of the most glucocorticoid sensitive (S) and most glucocorticoid resistant (R), excluding R11_m, healthy subjects one month after the very-low dexamethasone suppression test……………………...4

Supplementary Figure 2: The SAM curve of the metabolic profile data of the S (Group B) compared to the R (Group A) groups………………………………………………………………….5

Supplementary Table 1: The normalized blood plasma metabolomic dataset of the 22 participants used in multivariate statistical analysis

Peak Name	Metabolite Name	RT	Marker Ion	Chemical Category*	S1_f	S2_f	S3_m	S4_f	S5_m	S6_f	S7_f	S8_m	S9_m	S10_m	S11_m	R1_f	R2_f	R3_f
				Sex:	F	F	M	F	M	F	F	M	M	M	M	F	F	F
				Status:	S	S	S	S	S	S	S	S	S	S	S	R	R	R
1-monopalmitin	1-monopalmitin	39.359	371	1	0.051	0.206	0.239	0.200	0.126	0.233	0.228	0.086	0.125	0.283	0.241	0.201	0.223	0.162
2-hydroxybutanoic acid 2TMS	2-hydroxybutanoic acid	9.974	191+117	1	0.009	0.024	0.024	0.080	0.119	0.017	0.038	0.010	0.011	0.019	0.008	0.038	0.211	0.017
aminomalonic acid 3TMS	aminomalonic acid	19.564	218	3	0.027	0.036	0.041	0.029	0.023	0.037	0.031	0.021	0.026	0.039	0.017	0.035	0.036	0.023
benzoate 1TMS	benzoate	15.183	179	1	0.004	0.004	0.005	0.003	0.005	0.003	0.006	0.004	0.004	0.003	0.003	0.004	0.004	0.005
[illegible]	[illegible]	[illegible]	[illegible]	[illegible]	[illegible]	[illegible]	[illegible]	[illegible]	[illegible]	[illegible]	[illegible]	[illegible]	[illegible]	[illegible]	0.003	0.003	0.002	0.002
cho[illegible]	[illegible]	[illegible]	[illegible]	[illegible]	[illegible]	[illegible]	[illegible]	[illegible]	[illegible]	[illegible]	[illegible]	[illegible]	[illegible]	[illegible]	0.096	0.073	0.093	0.053
citr[illegible]	[illegible]	[illegible]	[illegible]	[illegible]	[illegible]	[illegible]	[illegible]	[illegible]	[illegible]	[illegible]	[illegible]	[illegible]	[illegible]	[illegible]	0.071	0.126	0.148	0.091
eryt[illegible]	[illegible]	[illegible]	[illegible]	[illegible]	[illegible]	[illegible]	[illegible]	[illegible]	[illegible]	[illegible]	[illegible]	[illegible]	[illegible]	[illegible]	0.002	0.004	0.003	0.003
eryt[illegible]	[illegible]	[illegible]	[illegible]	[illegible]	[illegible]	[illegible]	[illegible]	[illegible]	[illegible]	[illegible]	[illegible]	[illegible]	[illegible]	[illegible]	0.002	0.004	0.002	0.003
etha[illegible]	[illegible]	[illegible]	[illegible]	[illegible]	[illegible]	[illegible]	[illegible]	[illegible]	[illegible]	[illegible]	[illegible]	[illegible]	[illegible]	[illegible]	0.007	0.004	0.004	0.005
fum[illegible]	[illegible]	[illegible]	[illegible]	[illegible]	[illegible]	[illegible]	[illegible]	[illegible]	[illegible]	[illegible]	[illegible]	[illegible]	[illegible]	[illegible]	0.002	0.002	0.003	0.001
gala[illegible]	[illegible]	[illegible]	[illegible]	[illegible]	[illegible]	[illegible]	[illegible]	[illegible]	[illegible]	[illegible]	[illegible]	[illegible]	[illegible]	[illegible]	0.029	0.040	0.016	0.036
gluc[illegible]	[illegible]	[illegible]	[illegible]	[illegible]	[illegible]	[illegible]	[illegible]	[illegible]	[illegible]	[illegible]	[illegible]	[illegible]	[illegible]	[illegible]	6.981	8.570	6.821	8.519
glyc[illegible]	[illegible]	[illegible]	[illegible]	[illegible]	[illegible]	[illegible]	[illegible]	[illegible]	[illegible]	[illegible]	[illegible]	[illegible]	[illegible]	[illegible]	0.003	0.005	0.005	0.004
glyc[illegible]	[illegible]	[illegible]	[illegible]	[illegible]	[illegible]	[illegible]	[illegible]	[illegible]	[illegible]	[illegible]	[illegible]	[illegible]	[illegible]	[illegible]	0.020	0.029	0.045	0.024
glyc[illegible]	[illegible]	[illegible]	[illegible]	[illegible]	[illegible]	[illegible]	[illegible]	[illegible]	[illegible]	[illegible]	[illegible]	[illegible]	[illegible]	[illegible]	0.114	0.044	0.055	0.034
glyc[illegible]	[illegible]	[illegible]	[illegible]	[illegible]	[illegible]	[illegible]	[illegible]	[illegible]	[illegible]	[illegible]	[illegible]	[illegible]	[illegible]	[illegible]	0.130	0.159	0.177	0.145
lact[illegible]	[illegible]	[illegible]	[illegible]	[illegible]	[illegible]	[illegible]	[illegible]	[illegible]	[illegible]	[illegible]	[illegible]	[illegible]	[illegible]	[illegible]	0.283	0.332	0.343	0.251
lino[illegible]	[illegible]	[illegible]	[illegible]	[illegible]	[illegible]	[illegible]	[illegible]	[illegible]	[illegible]	[illegible]	[illegible]	[illegible]	[illegible]	[illegible]	0.002	0.004	0.009	0.003
myo[illegible]	[illegible]	[illegible]	[illegible]	[illegible]	[illegible]	[illegible]	[illegible]	[illegible]	[illegible]	[illegible]	[illegible]	[illegible]	[illegible]	[illegible]	0.022	0.045	0.041	0.038
myr[illegible]	[illegible]	[illegible]	[illegible]	[illegible]	[illegible]	[illegible]	[illegible]	[illegible]	[illegible]	[illegible]	[illegible]	[illegible]	[illegible]	[illegible]	0.001	0.002	0.007	0.002
octa[illegible]	[illegible]	[illegible]	[illegible]	[illegible]	[illegible]	[illegible]	[illegible]	[illegible]	[illegible]	[illegible]	[illegible]	[illegible]	[illegible]	[illegible]	0.030	0.044	0.084	0.026
oxa[illegible]	[illegible]	[illegible]	[illegible]	[illegible]	[illegible]	[illegible]	[illegible]	[illegible]	[illegible]	[illegible]	[illegible]	[illegible]	[illegible]	[illegible]	0.015	0.023	0.019	0.029
pho[illegible]	[illegible]	[illegible]	[illegible]	[illegible]	[illegible]	[illegible]	[illegible]	[illegible]	[illegible]	[illegible]	[illegible]	[illegible]	[illegible]	[illegible]	0.013	0.006	0.009	0.010
pyr[illegible]	[illegible]	[illegible]	[illegible]	[illegible]	[illegible]	[illegible]	[illegible]	[illegible]	[illegible]	[illegible]	[illegible]	[illegible]	[illegible]	[illegible]	0.022	0.024	0.024	0.035
pyru[illegible]	[illegible]	[illegible]	[illegible]	[illegible]	[illegible]	[illegible]	[illegible]	[illegible]	[illegible]	[illegible]	[illegible]	[illegible]	[illegible]	[illegible]	0.003	0.003	0.004	0.003
seri[illegible]	[illegible]	[illegible]	[illegible]	[illegible]	[illegible]	[illegible]	[illegible]	[illegible]	[illegible]	[illegible]	[illegible]	[illegible]	[illegible]	[illegible]	0.012	0.011	0.011	0.013
sorb[illegible]	[illegible]	[illegible]	[illegible]	[illegible]	[illegible]	[illegible]	[illegible]	[illegible]	[illegible]	[illegible]	[illegible]	[illegible]	[illegible]	[illegible]	0.026	0.048	0.029	0.036
suc[illegible]	[illegible]	[illegible]	[illegible]	[illegible]	[illegible]	[illegible]	[illegible]	[illegible]	[illegible]	[illegible]	[illegible]	[illegible]	[illegible]	[illegible]	0.001	0.001	0.001	0.001
thre[illegible]	[illegible]	[illegible]	[illegible]	[illegible]	[illegible]	[illegible]	[illegible]	[illegible]	[illegible]	[illegible]	[illegible]	[illegible]	[illegible]	[illegible]	0.006	0.007	0.011	0.009
thre[illegible]	[illegible]	[illegible]	[illegible]	[illegible]	[illegible]	[illegible]	[illegible]	[illegible]	[illegible]	[illegible]	[illegible]	[illegible]	[illegible]	[illegible]	0.032	0.033	0.025	0.017
Uh_[illegible]	[illegible]	[illegible]	[illegible]	[illegible]	[illegible]	[illegible]	[illegible]	[illegible]	[illegible]	[illegible]	[illegible]	[illegible]	[illegible]	[illegible]	0.007	0.012	0.011	0.012
Uh_[illegible]	[illegible]	[illegible]	[illegible]	[illegible]	[illegible]	[illegible]	[illegible]	[illegible]	[illegible]	[illegible]	[illegible]	[illegible]	[illegible]	[illegible]	0.037	0.042	0.047	0.040
Uh_[illegible]	[illegible]	[illegible]	[illegible]	[illegible]	[illegible]	[illegible]	[illegible]	[illegible]	[illegible]	[illegible]	[illegible]	[illegible]	[illegible]	[illegible]	0.263	0.180	0.324	0.170
Uh_[illegible]	[illegible]	[illegible]	[illegible]	[illegible]	[illegible]	[illegible]	[illegible]	[illegible]	[illegible]	[illegible]	[illegible]	[illegible]	[illegible]	[illegible]	0.047	0.110	0.086	0.159
Uh_[illegible]	[illegible]	[illegible]	[illegible]	[illegible]	[illegible]	[illegible]	[illegible]	[illegible]	[illegible]	[illegible]	[illegible]	[illegible]	[illegible]	[illegible]	0.013	0.031	0.027	0.014
Uh_[illegible]	[illegible]	[illegible]	[illegible]	[illegible]	[illegible]	[illegible]	[illegible]	[illegible]	[illegible]	[illegible]	[illegible]	[illegible]	[illegible]	[illegible]	0.003	0.006	0.005	0.004
Uh_[illegible]	[illegible]	[illegible]	[illegible]	[illegible]	[illegible]	[illegible]	[illegible]	[illegible]	[illegible]	[illegible]	[illegible]	[illegible]	[illegible]	[illegible]	0.014	0.025	0.023	0.012
Uh_[illegible]	[illegible]	[illegible]	[illegible]	[illegible]	[illegible]	[illegible]	[illegible]	[illegible]	[illegible]	[illegible]	[illegible]	[illegible]	[illegible]	[illegible]	0.004	0.006	0.004	0.006
Uh_[illegible]	[illegible]	[illegible]	[illegible]	[illegible]	[illegible]	[illegible]	[illegible]	[illegible]	[illegible]	[illegible]	[illegible]	[illegible]	[illegible]	[illegible]	0.018	0.009	0.007	0.017
Uh_[illegible]	[illegible]	[illegible]	[illegible]	[illegible]	[illegible]	[illegible]	[illegible]	[illegible]	[illegible]	[illegible]	[illegible]	[illegible]	[illegible]	[illegible]	0.001	0.002	0.002	0.002
Uh_[illegible]	[illegible]	[illegible]	[illegible]	[illegible]	[illegible]	[illegible]	[illegible]	[illegible]	[illegible]	[illegible]	[illegible]	[illegible]	[illegible]	[illegible]	0.038	0.067	0.061	0.062
Uh_[illegible]	[illegible]	[illegible]	[illegible]	[illegible]	[illegible]	[illegible]	[illegible]	[illegible]	[illegible]	[illegible]	[illegible]	[illegible]	[illegible]	[illegible]	0.001	0.003	0.004	0.003
Uh_[illegible]	[illegible]	[illegible]	[illegible]	[illegible]	[illegible]	[illegible]	[illegible]	[illegible]	[illegible]	[illegible]	[illegible]	[illegible]	[illegible]	[illegible]	0.068	0.077	0.085	0.075
Uh_[illegible]	[illegible]	[illegible]	[illegible]	[illegible]	[illegible]	[illegible]	[illegible]	[illegible]	[illegible]	[illegible]	[illegible]	[illegible]	[illegible]	[illegible]	0.096	0.083	0.089	0.058
Uh_[illegible]	[illegible]	[illegible]	[illegible]	[illegible]	[illegible]	[illegible]	[illegible]	[illegible]	[illegible]	[illegible]	[illegible]	[illegible]	[illegible]	[illegible]	0.005	0.013	0.013	0.005
Uh_[illegible]	[illegible]	[illegible]	[illegible]	[illegible]	[illegible]	[illegible]	[illegible]	[illegible]	[illegible]	[illegible]	[illegible]	[illegible]	[illegible]	[illegible]	0.002	0.007	0.007	0.004
Uh_[illegible]	[illegible]	[illegible]	[illegible]	[illegible]	[illegible]	[illegible]	[illegible]	[illegible]	[illegible]	[illegible]	[illegible]	[illegible]	[illegible]	[illegible]	0.001	0.001	0.001	0.001
Uh_[illegible]	[illegible]	[illegible]	[illegible]	[illegible]	[illegible]	[illegible]	[illegible]	[illegible]	[illegible]	[illegible]	[illegible]	[illegible]	[illegible]	[illegible]	2.629	4.340	4.459	4.342
ure[illegible]	[illegible]	[illegible]	[illegible]	[illegible]	[illegible]	[illegible]	[illegible]	[illegible]	[illegible]	[illegible]	[illegible]	[illegible]	[illegible]	[illegible]	0.524	0.794	0.352	1.299

No[illegible] included in published reports

* C[illegible]

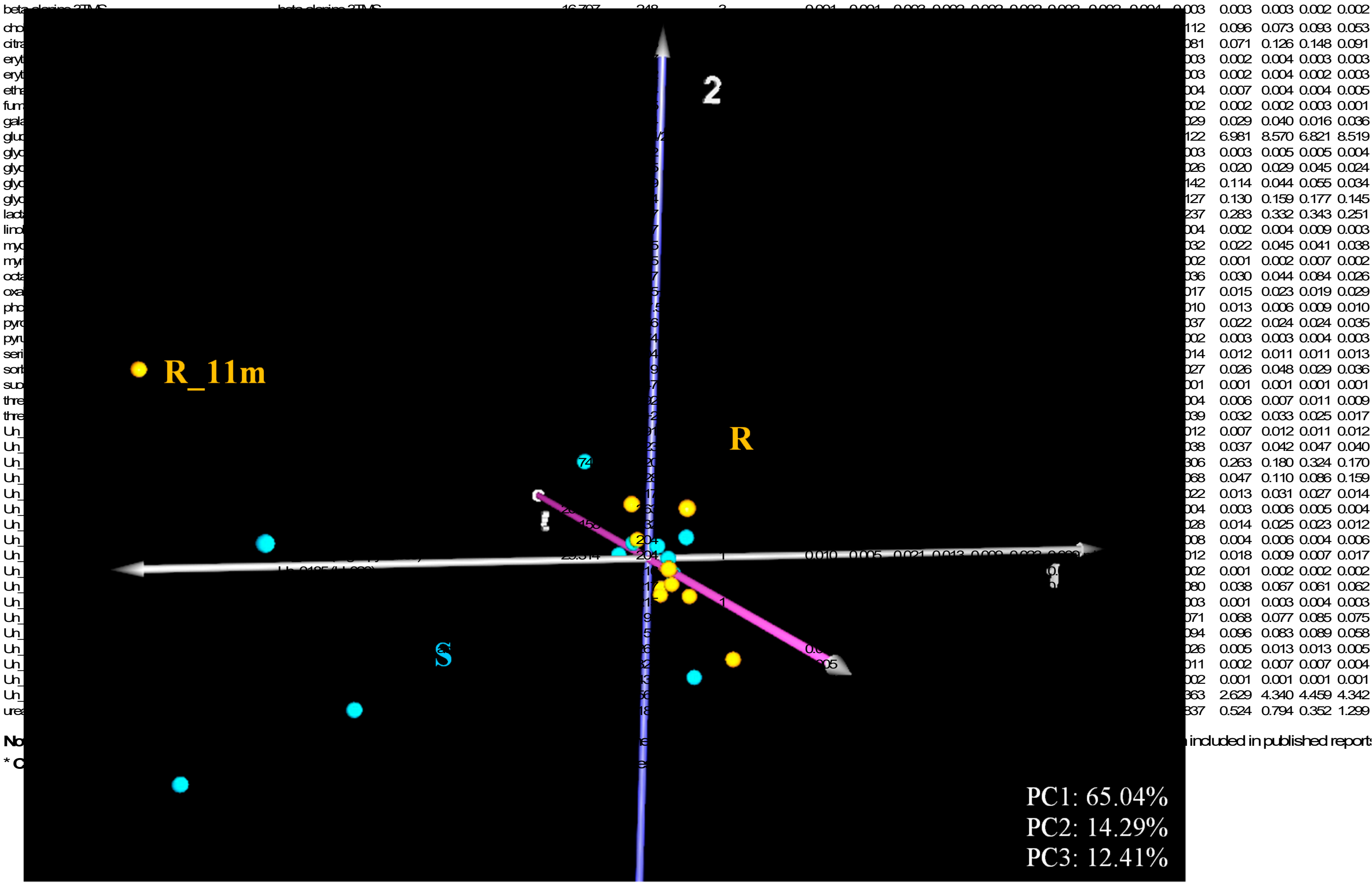

Supplementary Figure 1: The PCA graph of the metabolomic dataset of all 22 samples. The profile of the glucocorticoid resistant participant 11 (male; R_11m) exhibits a substantially different metabolic profile from all other samples (see also Suppl. Table 1) and has been excluded from further analysis. R (yellow) and S (light blue) depict, respectively, the plasma metabolic profiles of the glucocorticoid resistant and glucocorticoid sensitive participants.

Supplementary Table 2: Clinical characteristics, biochemical and endocrinological parameters of the most glucocorticoid sensitive (S) and most glucocorticoid resistant (R), in the absence of R11_m, healthy subjects one month after the very-low dexamethasone suppression test.

	**Glucocorticoid Sensitive (S), n=11**	**Glucocorticoid Resistant (R) but R11_m, n=10**	***p-value***
**Age (years)**	25.3 ± 3.9	27.5 ± 6.7	0.478
**Weight (kg)**	62.8 ± 12.3	60.5 ± 12.9	0.646
**Height (cm)**	1.7 ± 0.1	1.7 ± 0.1	0.276
**BMI (kg/m²)**	21.1 ± 2.0	21.6 ± 2.5	0.944
**25-Hydroxy-Vitamin D (ng/mL)**	16.0 ± 7.9	13.1 ± 5.3	0.652
**ACTH (pg/mL)**	33.2 ± 18.8	27.4 ± 16.3	0.459
**Androstenedione (ng/mL)**	2.9 ± 0.9	3.2 ± 1.2	0.748
**Anti-TG (IU/mL)**	20 ± 0.0	20 ± 0.0	0.999
**Anti-TPO (IU/mL)**	10.4 ± 0.7	11.1 ± 2.6	0.652
**ApoA1 (mg/dl)**	158.4 ± 8.0	167.6 ± 15.0	0.237
**ApoB (mg/dl)**	75.5 ± 14.4	71.4 ± 7.7	0.515
**Total Cholesterol (mg/dl)**	157.4 ± 16.9	156. ± 15.0	0.965
**Cortisol (nmol/L)**	664.2 ± 148.5	502.6 ± 286.0	0.270
**DHEAS (μg/dL)**	238.6 ± 146.0	264.6 ± 107.6	0.308
**E2 (pg/mL)**	103.0 ± 106.4	63.3 ± 78.1	0.857
**FSH (mUI/mL)**	5.2 ± 2.7	4.0 ± 2.3	0.246
**FT4 (ng/dL)**	1.1 ± 0.1	1.1 ± 0.1	0.484
**Glucose (mg/dl)**	73.2 ± 6.3	74.7 ± 13.6	0.965
**HDL (mg/dl)**	49.5 ± 7.0	52.9 ± 8.1	0.315
**IGFBP-3 (μg/mL)**	5.3 ± 1.0	5.1 ± 1.3	0.418
**IGF-I (ng/mL)**	259.2 ± 79.5	256.5 ± 68.1	0.857
**Insulin (μUI/mL)**	6.7 ± 2.7	14.3 ± 14.6	0.114
**LDL (mg/dl)**	90.7 ± 17.8	87.6 ± 13.5	0.762
**LH (mUI/mL)**	10.1 ± 14.9	6.4 ± 2.3	0.802
**Lpa (mg/dl)**	21.8 ± 37.4	25.8 ± 27.3	0.460
**Prolactin (ng/mL)**	24.9 ± 8.8	22.6 ± 8.8	0.275
**PTH (pg/mL)**	34.1 ± 15.2	38.9 ± 18.8	0.548
**SHBG (nmol/L)**	65.1 ± 27.9	45.5 ± 16.2	0.173
**T3 (ng/dL)**	102.3 ± 27.6	98.4 ± 21.8	1
**Testosterone (ng/dL)**	187.8 ± 231.6	292.2 ± 280.8	0.478
**Triglycerides (mg/dl)**	69.4 ± 30.0	74.2 ± 16	0.315
**TSH (μUI/mL)**	2.8 ± 0.9	2.1 ± 1.1	0.131

Note*:* The parameters for the two groups are expressed as mean ± SD.

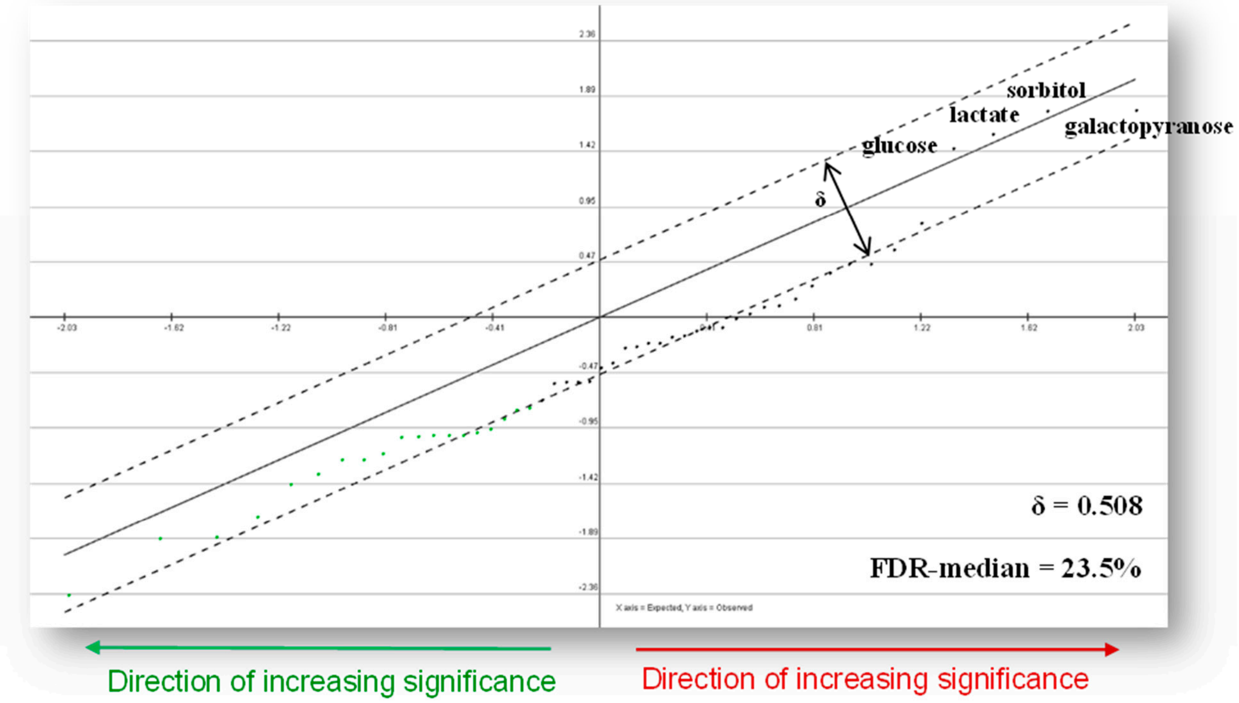

Supplementary Fig. 2: **The SAM curve of the metabolic profile data of the S (Group B) compared to the R (Group A) groups.** Each point corresponds to a particular metabolite. The axes of the graph correspond to the observed (y axis) and the expected (x axis) values calculated by the method for the analyzed metabolites; the expected value refers to the case in which the difference in the metabolite abundance between the two compared groups would have been based only on random error. The dashed lines define the strictest threshold of significance δ for which significant metabolites were obtained in this study; FDR (median) depicts the false discovery rate (%) for the particular δ. If the difference between the measured and the expected value for a metabolite concentration is in absolute value larger than δ, then if it is positive or negative, this metabolite is, respectively, identified of significantly higher (red) or lower (green) concentration in the S compared to the R group. The further a significant metabolite is from the origin (0,0) of the graph, the higher the statistical significance of the metabolite. The list of the negatively significant metabolites depicted as green points in this figure is shown in Table 3 in decreasing significance. Here indicated are the four metabolites, which exhibit higher abundance in the S compared to the R groups, but are just below the positive significance threshold. Finally, the negative intercept of the SAM curve indicates an overall lower abundance of all 50 analyzed metabolites in the S compared to the R group.
